# A Practical Treatise on Inflammation, Ulceration and Induration of the Neck of the Uterus—with Remarks on the Value of Leucorrhœa and Prolapsus Uteri, as Symptoms of Uterine Disease

**Published:** 1847-10

**Authors:** 


### PART II.—REVIEWS.

### ARTICLE VIII.

*A Practical Treatise on Inflammation, Ulceration and Induration of the Neck of the Uterus—with Remarks on the value of Leucorrhœa and Prolapsus Uteri, as symptoms of uterine disease.* By JAMES HENRY BENNETT, M. D., Licentiate of the Royal College of Physicians, etc., etc. Philadelphia: Lea & Blanchard. 1847. pp. 146.

We have already had occasion to express our favorable opinion of the value of this work, and announced a determination to return to the subject and present our readers with such copious extracts as would not only enable our readers to form an estimate of its merits, but aid them in the treatment of those forms of uterine disease which fall to the lot of every practitioner. In pursuance of this plan, we now proceed to extract some of the most important parts, hoping thereby to induce physicians to possess themselves of the book itself, than which a more useful little volume could scarcely be added to their libraries.

The healthy state of the cervix is thus described at the opening of the first chapter.

"In the healthy condition, the cervix uteri is perfectly soft and smooth. On being pressed by the finger, no hardness or resistance, indicating condensation of tissue, is felt. There is at the same time a certain degree of elasticity about it, the varying degree of which indicates general or local congestion, or atony of the uterine system—states which, however, as Dr. Ashwell justly remarks, can only be appreciated by long habit. In the healthy condition, the surface of the neck of the uterus is generally unctuous to the touch. The layer of mucus by which it is then covered accounts for this very characteristic sensation. There is also complete absence of pain on pressure. In examining the cervix by the toucher, it is advisable to appreciate carefully the state of the entrance to its cavity, as slight local induration existing on or within the margin of the lips might otherwise escape notice. The pulp of the finger should be brought successively to bear on each part of the surface of the organ, above, below, and on each side, which may be easily accomplished. Not only does this mode of examination contribute to render our sensations of density and smoothness more perfect, but it also enables us to judge of the size and freedom from adhesions of the body of the uterus itself. In the unimpregnated state, and when not morbidly enlarged, the body of the uterus moves readily on pressure being made on the neck; pressure thus applied, acting as on one extremity of a lever—that is, raising the other in the opposite direction. If these facts respecting the healthy uterine neck are borne in mind, the detection of disease becomes comparatively easy."

Proceeding to the symptoms of the disease, he says—

"SYMPTOMS.—When inflammation attacks the cervix uteri in women who have not conceived, it is nearly always confined to the mucous membrane, the deeper structures seldom becoming implicated, except in cases of general metritis. The inflammation may co-exist with general vaginitis, as is usually the case in gonorrhœa; it may be confined to the uterine neck, and to that part of the vaginal cavity which is in contact with it—viz., the superior fourth or fifth; or it may be limited to the orifice of the os uteri. The leucorrhœal discharge may be a prominent symptom, or it may be absent, or nearly so; which is the case when the inflammation is very limited, the mucoso-purulent secretion being then but slight, and lost in the vagina. This generally occurs when the inflammation is the result of sexual communication. There are, however, other symptoms present to guide us in our diagnosis. The patient complains of pain in the loins, and sometimes, of deeply-situated pain in the hypogastric region, behind the pubis, and, a most important symptom, intercourse is painful. This fact alone may lead us to suspect the existence of disease. Sometimes there is a vivid perception of heat at the superior portion of the vagina. There is no sensation of weight, heaviness, or bearing down, except in extreme cases, in which the malady has been long neglected.

*Toucher*.—On examining by the toucher, the neck of the uterus is found hotter than the lower part of the vagina; it has lost its unctuous, greasy feel; its volume is more or less increased, as also its elasticity, owing to its being more or less congested. Still there is no general or deep-seated induration of its tissue. The surface, likewise, is smooth and unresisting, unless ulceration has set in. When this is the case, it is at the orifice of the uterine cavity that the ulceration commonly begins, and from that region that it spreads; owing, no doubt, to the great tenuity and delicacy of the mucous membrane. Pathologists generally state that the ulceration may be recognized, by its producing the sensation that a velvety surface would offer when the finger is passed lightly over it. Finding, however, that this peculiar sensation is so difficult to appreciate in this form of the disease, that those who rely upon it alone must be as often wrong as right, I have endeavored to discover a more correct guide, and have ascertained that ulceration of the mucous surface, however limited, almost invariably gives rise to slight induration of the tissue underneath, which induration is very perceptible to the touch. In the form of ulceration that we are now examining, the induration to which I allude is quite superficial, not extending to the central tissue of the uterine neck. It is merely a thickening of the ulcerated mucous membrane, and of the sub-cellular tissue, most perceptible at the circumference of the ulceration; yet it is easily appreciated by the finger of one who is accustomed to look for it, and to him it is a valuable symptom. This superficial induration is generally felt most distinctly at the edge of the uterine lips, where the mucous membrane passes into the cavity of the neck, and where, consequently, two mucous thicknesses are approximated by the folding of the membrane. Although I have found this symptom of great assistance in the diagnosis of ulcerations, I must confess, nevertheless, that it is not infallible. In the very first stage of ulceration, induration may not yet exist, whilst, on the other hand, the ulceration may heal, and the superficial induration remain for a few days. When the inflammatory induration extends to the entire substance of the cervix, as it generally does if the ulceration exists in women who have had children, the superficial induration is necessarily lost in the general hardness. Pressure on the inflamed and ulcerated cervix will often, not always, occasion slight pain, which is never the case in the healthy state.

"There being, thus, great difficulty in arriving at a satisfactory diagnosis by means of the toucher alone, it is generally necessary to resort to the speculum, in order to ascertain correctly the true state of things, its use being calculated to remove all doubts as to the state of the parts. This remark applies not only to those who are unaccustomed to the treatment of uretine diseases, but even to those whose touch has been fully educated.

"*Speculum.*—On examination by the speculum, a certain quantity of mucoso-purulent matter is always found at the superior region of the vagina, even when the lining membrane of that organ is not inflamed; the cervix uteri is generally increased in size, but seldom so much so as not to be admitted into the cavity of an ordinary sized conical speculum, the one I generally use, and by far the most convenient and the least painful to the patient. The tumefaction is mostly greatest on the upper lip, which is the larger one of the two in the healthy condition; it is therefore often necessary, in order to expose the orifice of the os, to raise the speculum towards the pubis, and by thus slightly pressing with the superior edge of the instrument on the anterior lip, to push it back, and allow the inferior one to enter its cavity. Even if the cervix uteri is too large to be admitted at once into the speculum, by thus alternately depressing its different parts, the entire organ may successively be brought fairly into view. When inflamed, the tumefied cervix presents a more or less intense red, glistening hue, instead of the pale, dull, whitish colour, which is natural to it. On its surface may frequently be seen small white or red vesicular, or papular, elevations, the result of distention of the mucous follicles, or of their hypertrophy. Different forms of inflammation have been admitted by some writers, founded on this appearance, but without any practical utility whatever. When the mucous membrane is ulcerated, the glossy appearance of the membranous surface is lost, and a number of vascular granulations, of a vivid red hue, are seen covering the ulcerated region, after the mucus has been wiped away with a pledget of lint—a necessary precaution. Sometimes the ulcerated surface appears raised above the adjacent level, whilst occasionally, on the contrary, it appears depressed. When the ulceration is at the entrance of the os uteri it is often difficult to discover, unless the uterine lips be slightly separated. There is generally a mass of semi-transparent mucus occupying the cavity of the os uteri. The ulceration may be so superficial and slight as to be scarcely perceptible, or extend over a considerable portion of the cervix. In many cases, the pressure of the edge of the speculum, or even of the pledget with which the mucus is wiped off, occasions a slight oozing of blood from the abraded or ulcerated surface. This also frequently occurs when patients thus affected expose themselves to intercourse—a fact of which they themselves are often cognizant. Menstruation is generally more painful than in the healthy state, owing to the temporary congestion of the uterus increasing the inflammatory irritation of the cervix. Indeed, the occurrence of the various symptoms of painful and difficult menstruation, when coupled with a leucorrhœal discharge, may be considered, in most cases, as pathognomonic of the inflammation and ulceration of the cervix. Occasionally, slight irritation of the urinary organs is present, giving rise to frequent desire to urinate. The annoyance and distress of mind which the local symptoms sometimes produce, coupled with the leucorrhœal discharge, when it is abundant, may react more or less on the general health, and give rise to dispepsia, palpitation, general weakness, &c.

"Such are the symptoms which ulceration of the cervix and os uteri usually occasion in the unimpregnated female. The inflammation, ulceration, and induration, are nearly always superficial—limited to the mucous membrane. The cervix becomes tumified, congested, but remains soft and spongy. There is scarcely ever the deep-seated, solid engorgement of the cervix, which is so often met with as the result of the same lesions in females who have borne children, and which is occasioned by inflammation and effusion of lymph in the central tissues of the neck, giving rise to the peculiarly distressing bearing-down pains experienced by persons thus afflicted. The reason is evident. Although subject to the periodical menstrual congestion, the uterus is, until impregnated, in a dormant state, as it were. Its mucous membrane is a mere film, and its proper tissue, which we have followed into the neck, is in an elementary fibro-muscular state, very sparingly supplied with blood, and possessing a very subdued vitality. It is owing to these anatomical and physiological circumstances, in my opinion, that the inflammations and ulcerations of the cervix uteri *seldom* assume the more serious form which I shall have to describe as that which is frequently met with in women who have borne children. I shall conclude the above brief account of this species of uterine inflammation, by the narration of two cases, selected from many, which will admirably illustrate the facts that I have above stated."

We add one of the cases given in illustration.

"*Disease rather severe.—Cause, marriage.—Cure perfect.*

"At the beginning of 1844, a gentleman, who had been married about four months, requested me to see his lady, who had, he stated, been suffering for some time. The lady, four-and-twenty years of age, was apparently in the enjoyment of robust health, the various functions being all accomplished with great regularity. On inquiring minutely, however, into her state, I found that she had experienced pains in the loins nearly ever since her marriage ; that these pains had gradually increased, had lately been accompanied by slight pain behind the pelvis, and by a deep-seated sensation of heat in the same region ; that intercourse, at first unattended by pain, had, a few weeks after marriage, become painful, and was then unbearable, from the last mentioned cause. There was no perceptible leucorrhœal discharge. Being convinced that inflammation and ulceration of the uterine neck were the cause of these symptoms, I obtained the consent of the parties to an examination.

"On practising the toucher, I found increased heat in the superior region of the vagina, and a large tumefied, but soft and pulpy, cervix uteri. The anterior lip was evidently much more tumefied than the posterior; on its margin, I distinctly felt a superficial induration of several lines in length, presenting a rather uneven surface. The speculum having been introduced, I found the mucous membrane of the lower two-fourths of the vagini perfectly healthy, but the superior fourth was red, inflamed, and partly covered with a mucoso-purulent secretion, especially where in contact with the inflamed cervix. The latter was of an uniform red color. The upper lip was so much congested and swollen, as to occupy nearly all the concavity of the speculum, and to cover the orifice of the uterine cavity, and the under lip. On its being pushed back so as to expose the latter parts, a circular ulceration, about the size of a shilling, was discovered around the os, but more especially extending on the anterior lip. The pressure of the speculum was found rather painful. A slight oozing of blood took place on the copious mucoso-purulent secretion, which covered the ulcerated surface, being wiped away. When this had been done, the mucus passing from the interior to the cavity of the neck was found quite transparent, a proof that the internal surface of the uterine cavity was not inflamed. The entire surface of the cervix, and upper part of the vagina, was painted over with the solid nitrate of silver, which was passed two or three times over the ulcerated region, and into the cavity of the os for a couple of lines. The application of the caustic was scarcely attended with any pain. The patient was then told to use cold water vaginal injections several times a day, for two days, and after that period, injections with the sulphate of zinc. She was also requested to remain quietly at home, on an easy chair, or a sofa, and, as a matter of course, forbidden any communication with her husband.

"A couple of days after the cauterization, the pains in the loins and pelvis had much abated, as also the other symptoms above mentioned.

"On the eighth day, the cauterization was repeated, the tumefaction of the cervix had much diminished, as also the inflammatory congestion. The ulcerated surface was decidedly smaller. The same local treatment was pursued. On the sixteenth day, nearly all pain in the loins had disappeared; the cervix uteri was evidently rapidly regaining its natural size, and the ulceration had still further diminished. She was allowed to ride out in a carriage, and even to walk with moderation.

"Cauterization with the nitrate of silver was again resorted to on the twenty-first and twenty-fifth day, but much more slightly, and on the thirty-second day she was quite cured. The ulceration, had cicatrized without leaving the slightest induration behind it. The tumefaction of the uteine neck had disappeared, and it had regained its usual coloration and unctuous feel to the touch. I need scarcely say that not a vestige of the symptoms experienced during the preceding months remained. I gave her no medicine internally during the treatment, because she did not require any, and did not even think it necessary to modify her usual diet, which was simple."

Chap. II. is devoted to the consideration of the disease in those who are pregnant, or who have borne children; the changes induced by this state being thus set down, as follows:

"*Physiological and Anatomical considerations.—Causes, symptoms, progress.—Illustrative cases.*

"In the previous chapter, I have examined the causes and symptoms of the inflammation of the uterine neck in females who have never borne children. I have stated that in them the cervix uteri, as well as the uterus itself, may be considered in a dormant state. We will now proceed to the study of inflammation, ulceration, and induration of the cervix uteri in women who are pregnant, or have borne children, by far the most important part of the task which I have undertaken.

"As soon as conception has taken place, a new life, as it were, dawns on the uterus and its appendages. Instead of remaining in a quiescent condition, merely disturbed at periodical intervals by the menstrual congestion, the uterus assumes a high degree of vitality, becomes the seat of a most active nutrition, and rapidly increases in size. The hard fibro-muscular tissue of which it is formed undergoes, apparently, a complete transformation, and assumes the decided characteristics of muscular structure; the arteries and veins, previously so small as to be followed with difficulty, are developed to an enormous extent, and the entire organ becomes one of the most instead of one of the least vascular in the human economy. The cervix uteri participates in the change; it becomes turgid; it swells, softens, and its entire structure is modified by the exaggerated organic activity which pervades the uterine system. This nutritive activity gradually increases, until at last labour takes place, and the fœtus is expelled. The uterus then contracts on itself, and partially regains its former state; I say, partially, for it is well known that, as long as menstruation persists, the uterus of a woman who has conceived never returns entirely to the size which it presented previous to conception. It is larger, rather more muscular, and endowed with greater vitality; consequently, it is more liable to disease, and especially to inflammation. In confirmation of this fact I may mention a circumstance which I have repeatedly observed—viz., that in metritis unconnected with pregnancy, which is nearly always caused by the sudden suppression of the menstrual flux, the body of the uterus enlarges very much more in women who have borne children than in those who have not. In the latter, also, sudden suppression of menstruation gives rise very frequently to ovaritis, instead of metritis, which is very seldom the case with the former—an additional proof of the greater susceptibility to uterine inflammation of women who have borne children.

"This remark applies even more to the cervix uteri than to the body of that organ, as the cervix is naturally rather more vascular, possesses a little cellular tissue, which the uterus itself does not, and is covered with a mucous membrane, much thicker and much better organized than the one which lines the uterine cavity. This being the case, it stands to reason that inflammation of the cervix uteri will extend much more frequently throughout its entire substance, and present much greater gravity, in females in whom the uterus is thus modified, than it does in those in whom the uterus has not undergone any change. In the following remarks I shall endeavour to show that such is really the result produced by the structural modifications which follow conception. I shall begin by examining the causes which give rise to this form of the disease.

"CAUSES.—Out of twenty cases of non-venereal inflammation and ulceration of the cervix which we meet with in practice, seventeen may be directly traced to abortion or to labour, two will recognise other causes, and occur in women who have borne children, whilst one only will be found in females who have never conceived. I do not give this statement as the result of statistical researches, but as the impression left on my mind by the examination of a very large number of cases.

"When the disease is not the result of abortion or of labour, but occurs in women who have borne children, it may depend on the same causes as in women who have never conceived—causes which we have already enumerated, (sexual irritation, vaginitis, aphthæ, &c.;) or it may be the result of the localization, under a chronic form, of general metritis in the central tissues of the neck. When this takes place, the induration and hypertrophy are primary, and the ulceration secondary; the friction of the indurated cervix against the superior region of the vagina occasioning and keeping up a degree of irritation of the mucous membrane which often terminates in ulceration. This cause of ulceration of the cervix is, I believe, very rare in females who have never conceived, the central tissues of the uterine neck being in them partly protected against inflammation by the peculiar condition of its hard fibro-muscular structure.

"When inflammation and ulceration are the result of abortion or labour, they may recognize the same origin—general metritis, occasioned by the abortion or labour, localizing itself, under a chronic form, in the cervix, and giving rise, firstly, to hypertrophy, and subsequently, to ulceration. Indeed, many of the Paris physicians and surgeons appear to think that such is nearly always the case; that ulceration is, in most instances, the *result*, and not the *cause*, of general induration and hypertrophy of the cervix. With this opinion I cannot agree. I admit that some ulcerations are generated as above described; but I believe that they are the exceptions, and that, in the great majority of cases, the hypertrophy and general induration (engorgement) are caused and perpetuated by the presence of the superficial ulceration. This conviction is founded on my having generally perceived, in recent cases, that the extent and degree of the engorgement coincided with the extent and degree of the ulceration, and on my having been able repeatedly to follow the gradual increase of the inflammatory induration of the cervix coinciding exactly with that of the ulceration. On the other hand, in the cases in which the inflammation and ulceration of the mucus surface have caused the general induration, if the latter persists, it becomes an important cause of disease, continually reproducing the ulceration, unless means be taken to cure it, as well as the ulcerated surface.

"Even among those who recognize, like myself, the *ordinary* pre-existence of inflammation and ulceration of the surface of the cervix to general inflammatory induration of its body, erroneous views, I believe, exist. Thus, in a memoir published a few years ago in the 'Archives,' by M. Gosselin, a clever French surgeon, it is stated, without exclusive reference to the sequelæ of labour, that ulcerations of the uterine neck are almost invariably occasioned by internal metritis, or inflammation of the lining membrane of the uterine cavity, and that this form of inflammation nearly always accompanies and keeps up the ulceration. I am quite prepared to acknowledge that internal metritis, especially after labour, is an important cause of inflammation and ulceration of the cervix, as I shall presently explain, but I certainly cannot admit that it is nearly always the sole cause of the lesion, or that it generally coincides with it in its after stages. M. Gosselin founds his opinion principally on the mucoso-purulent character of the discharge issuing from the os uteri, and on the slight hypogastric pains which usually exist in such cases. These data are not however, in my opinion, sufficient to authorize his views. When ulceration of the cervix exists, its general seat is around the uteri, and it very often passes more or less into the cavity of the os. Now, when we consider that the cavity of the uterine neck extends some distance before it reaches that of the body of the uterus, it must be allowed that the presence of a little muco-pus between its lips is not a sufficient proof of an inflammatory secretion taking place in the interior of the uterus. If, however, a wide, thick stream of muco-pus issues deeply from between the lips, it probably originates in the uterine cavity, and the membrane which lines it is most likely inflamed. I have, however, not very often found, in ulceration of the cervix, that this is the case; and when I have, the hypogastric pains have always been much more severe than those which accompany even severe ulceration. I have generally, also, observed, in these latter cases, more or less febrile re-action. I myself believe that we may explain by other considerations the undeniable fact of abortion and labour giving rise to the great majority of the cases of ulceration and general hypertrophy of the cervix which we meet with in practice."

"Symptoms and Progress.—The *first* symptoms of inflammation and induration of the uterine neck in women who *have* borne children are the same as in those who have not, but in the former they very soon acquire an intensity which they seldom present in the latter. Moreover, in women who have borne children, owing to the greater vitality of the uterine tissue, as I have already explained, the inflammation readily extends to the central structure of the cervix, and gives rise to *inflammatory induration of the entire organ*, which induration is accompanied by a new train of symptoms. Thus it will be seen that the form of the inflammation and ulceration which is observed in women who have never conceived—is, in reality, merely the first stage of the disease, and it is found in females in whom the uterine structure has undergone the change which accompanies and follows impregnation. It would, indeed, be scarcely worth while to establish any distinction between what, in reality, are merely different stages of the same disease, were it not that such a distinction has a decided practical advantage. It impresses forcibly on the memory that, in one class of females, the incipient symptoms of the disease only are to be expected, and thus draws the attention to data which otherwise, in that case, would not, most likely, be deemed sufficiently important to demand investigation. It also enables us to understand at once how it is that in some cases very simple therapeutic means are nearly always successful; whereas in others these same means frequently fail to effect a cure, the disease requiring more energetic treatment.

"I need not, therefore, lay much stress on the symptoms which indicate *incipient* inflammation of the uterine neck, in women who have conceived, as I have already described them. They are slight lumbar and hypogastric pains, with or without leucorrhœal discharge. On examination by the toucher, heat of the upper part of the vagina, fulness, congestion of the cervix, absence of the unctuous sensation which the healthy cervix presents; when ulceration exists, a soft, velvety surface, resting on a very superficial induration. On examination with the speculum, redness of the cervix, which is more or less tumefied; the redness being uniform, or presenting red papulæ, or white pustulæ, constituted by mucous glands, hypertrophied, or distended with muco-pus. If ulceration exists, the ulceration is nearly always situated round the os uteri, and may present merely a scarcely perceptible abrasion, or a large ulcerated surface covered with numerous florid granulations: the ulceration is covered with muco-pus, and bleeds very readily.

"The induration which accompanies ulceration of the cervix does not, however, long remain confined to the surface in women who have borne children. The inflammation gradually extending to the deeper structures, a great portion, or the whole cervix, becomes more or less actively hypertrophied and indurated. That this inflammatory induration is only, in the great majority of cases, the result of the extension of the ulcerative inflammation, is, in my opinion, an undeniable fact. I have repeatedly been able to follow instances—such as that of Octavie, (case 3)— in which a slight ulceration was at first the only lesion, and in which the general induration subsequently made its appearance, gradually becoming more and more marked as the ulceration increased in extent. I have also repeatedly seen an ulceration confined to one lip, accompanied by engorgement of that lip only. Indeed, there is generally, in recent cases, a very evident conformity between the degree of the general induration and the extent and virulence of the ulceration. We must also take into consideration another very important circumstance—viz., the time that has occurred since the last abortion or labour. The nearer a female is to the epoch at which she was last delivered or miscarried, when attacked with inflammation and ulceration of the cervix, the greater will be the central engorgement produced by the ulceration. This hypertrophy and induration is generally confined to the cervix, but sometimes it passes on to the body of the uterus, then, obviously, likewise the seat of inflammation. At first, the central induration is evidently of an active inflammatory nature, as indicated by the increased heat of the organ, the vivid redness, and slight pain on pressure. If, however, it is not subdued in the course of time, these symptoms of inflammatory engorgement partially subside, and the organ becomes the seat of mere chronic hypertrophy, the inflammatory character of which is scarcely recognizable. The size of the engorged cervix varies from that of a walnut to that of an egg.

"The uterus is so slightly poised or suspended in the cavity of the pelvis, that the slightest modification in its volume gives rise to a change in its position. The inflammatory hypertrophy of the cervix increasing considerably the specific gravity of the inferior portion of the uterus, the entire organ descends, prolapses. The cervix is thus brought much nearer to the vulva: at the same time it frequently falls backwards, and presses on the posterior parietes of the vagina, whilst the body of the uterus is carried more or less forward. This latter change of position, which constitutes anteversion of the uterus, or retroversion of the neck, is not, however, so common as partial prolapsus. Whenever there is much engorgement of the cervix there is always more or less prolapsus if the patient is standing; the degree to which it is carried depending on the extent of the hypertrophy and on the state of the vagina. If the vagina has retained its tone and its contractility, it will support the uterus; but if, on the contrary, it is lax, and offers no support to the engorged cervix, as is sometimes the case in women who have had many children, the latter may fall as far as the orifice of the vulva. This abnormal laxity of the vagina may be occasioned by the disease itself; the distension of the superior portion of the vagina by the hypertrophied cervix diminishing its tonicity. The engorged cervix then falls, as it were, into a non-contractile pouch.

"The direction of the healthy cervix varies considerably, even in females who have never suffered from uterine disease. In most it is directed to the vulva, whereas in others it is turned backwards, and points to the anus. This latter direction of the cervix is stated, by M. Lisfranc, to be one of the results of marriage. It is easy to be understood, that in females with whom the cervix is naturally long, marriage should produce this effect. Whether the backward direction of the cervix be natural or acquired, it is certain that it constitutes a predisposition to anteversion of the uterus or retroversion of the cervix, should the latter subsequently become the seat of general induration.

"The condition of the patient is considerably modified by engorgement of the cervix, and the gravity of the disease much increased. The sensation of weight and heaviness in the hypograstic region, scarcely perceptible as long as the cervix is merely congested, becomes very marked and distressing, especially in walking and standing. Indeed, if the inflammatory hypertrophy is considerable, the patients not unfrequently complain that whenever they are on their legs they feel as if the womb were on the point of falling out of the pelvis. The deep-seated hypogastric pain is increased, and sometimes pressure above the pubes is slightly painful. The pain in the loins and in the lumbar region is generally continued, and most distressing. Severe pains are also often experienced in the thighs, along the course of the sciatic, obturator, and crural nerves. These pains are no doubt, in a great measure, to be attributed to pressure, and to the traction exercised by the engorged and prolapsed cervix on the nerves supplied to the uterus by the lumbo-sacral plexus. They are much more severe when the cervix is ulcerated and engorged, than when it is merely ulcerated. When there is retroversion of the neck, the hypertrophied cervix pressing on the rectum renders evacuation of the fæces difficult and painful. The body of the organ being also thrown forward, may irritate the bladder, and occasion frequent desire to urinate. The presence of the ulcerated and indurated cervix in the cavity of the vagina secreting an abundant muco-purulent fluid, which partly stagnates in that organ, is inevitably followed by the inflammation of its mucous membrane, and by general vaginitis, which increases the amount of the leucorrhœal discharge.

"When a patient is in this state, often long before, the general health begins to fail. Racked with pain, suffering from an abundant leucorrhœal discharge, it is impossible that the economy should not suffer. In nearly all cases, the appetite flags, the tongues become loaded, the bowels irregular, and in the more severe ones, the patient loses flesh and strength, suffers from continued headach, from want of sleep, and becomes dyspeptic, hysterical, hypochondriacal. As the disease gains ground, when proper measures are not adopted to arrest its progress, all these symptoms increase in intensity; the patient is nearly unable to leave her bed, and the skin assumes the yellow cadaverous hue, which is occasionally seen in severe chronic inflammatory disease of the uterine organs, and which may be mistaken, and no doubt occasionally is, for a symptom of cancerous cachexia. In these severe cases, the inflammation and induration are seldom, if ever, confined to the cervix uteri. They extend more or less to the body of the uterus, giving rise to a subacute form of metritis, indicated by the increased size of the organ, and by the increased severity of the uterine pains. There is also, generally speaking, more or less febrile reaction, especially in the latter part of the day.

"Whenever there is even superficial inflammation of the cervix uteri, menstruation is modified by its existence. Indeed, in slight cases, the modification may often materially assist the diagnosis. The monthly congestion of the uterus generally appears to exacerbate the local inflammation, which, on its side, renders the due performance of the menstrual excretion difficult, probably by abnormally increasing the uterine congestion; thence intense uterine pains, increased pain in the loins, and not unfrequently hysterical symptoms. This exacerbation often begins two or three days before the appearance of the menstrua, and lasts for one, two, or more days afterwards. Generally speaking, the ordinary duration of the menstruation is curtailed, and the amount of the excretion diminished; but it is not always so, for sometimes, more especially in severe cases, flooding will occur at each menstrual epoch, lasting many days. There is but little doubt that the monthly congestion instead of favoring the resolution of uterine inflammation, as some authors have pretended, is one of the chief causes of its being difficult to overcome.

"*Toucher.*—In this form of inflammation and ulceration of the uterine neck, the toucher is a much more valuable means of diagnosis than in the former, or, at least, gives much more certain data. The finger very soon reaches the prolapsed cervix, which is only one, two, or three inches from the vulva, especially if the woman is standing, instead of four or five, as is naturally the case. The vagina is moistened by an abundant leucorrhœal secretion, and is often hotter than usual. The increased size of the cervix is at once recognized, as also its resistance to pressure and great density. The os uteri is nearly always more or less open, so as to admit a small portion of the extremity of the finger, and the soft velvety sensation of the ulcerated surface is occasionally very evident, when the granulations are luxurious or fungous.

"Sometimes, if the disease is the result of difficult or instrumental labour, or of a miscarriage, the cervix is found deeply fissured, so as to present several lobes or lobules. When this occurs, even a practitioner who has had much experience in uterine disease, may be led to conclude that the affection is of a cancerous nature, unless he analyze very minutely the history and symptoms of the case; the more so, as it is in such instances that the general symptoms are the most alarming. I have met with several cases of this kind, in which so many of the symptoms of ulcerated uterine cancer were present, (cachetic cancerous tinge of the skin, extreme emaciation, abundant leucorrhœal discharge, often tinged with blood, occasional flooding, indurated, lobulated, and ulcerated cervix,) that it was with difficulty I could form a correct diagnosis. This may be done, however, if we attend to the history of the patient, and examine minutely the local state of the uterine organs. The origin of the disease may be always traced to difficult parturition.

The fissures divide the cervix into lobules, but each lobule is itself smooth and round, however indurated it may be. The fissures radiate from the os. The vagina is perfectly free at its union with the cervix, which is seldom the case in advanced ulcerated cancer. Moreover, in these extreme cases, the inflammation always extends deeply into the tissue of the uterus, the volume of which is increased. By pushing back the posterior or anterior cul de sac of the vagina with the pulp of the index finger, it is not difficult to ascertain whether or not the induration of the cervix extends to the posterior or anterior plane of the uterus. In order to ascertain whether the volume of the uterus itself is increased, one or two fingers of the right hand must be introduced into the vagina, the pulp of the fingers directed towards the pubis of the patient. The fingers being then placed underneath the cervix, and the posterior vaginal cul de sac pushed back, it is very possible to raise the uterus by them. If the left hand is placed at the same time on the hypogastric region immediately above the pubis, and the patient is told to relax the abdominal parietes, the abdomen may be depressed over the uterus, so as for the latter organ to be distinctly felt between the finger or fingers in the vagina, and the hand over the pubis. Its volume may thus be very accurately appreciated. The ovaries, lateral ligaments, indeed, the entire pelvic cavity, may be explored in this way with the greatest ease. It is, however, absolutely necessary, to accomplish this exploration satisfactorily, that the examination should be made whilst the patient is lying on her back. The two hands could not be used freely if the patient were placed on her side, as is usually the case in this country."

That ulcerations should be so frequent without having been at all suspected, will, we know, be surprising to some; but when we consider the various causes of irritation applied to these parts, the injuries and abuses to which they are subject, their existence can no longer be a matter of wonder. If any doubt the frequency of the forms thus far described by Dr. Bennett, they will probably be still more skeptical in relation to the occurrence of these ulcerations during pregnancy; but the following cases, and we might add another from our own experience, will show that pregnant women are by no means exempted from such disease.

"*Rather severe case, from the Thesis of M. Costilhes.*

"Clara B——, aged twenty-one, entered St. Lazare the 2d Sept., 1842, being in the fourth month of her first pregnancy. She has never had any syphilitic disease.—*Toucher*: neck voluminous, indurated, ulcerated, and sanguinolent; she has pain habitually in the hypogastrium.—*Speculum:* on the engorged cervix an ulceration the size of half-a-crown, of a fungous, vegetating nature, and violet coloured; abundant leucorrhœa.—*Treatment:* injections with decorations of walnut leaves; cauterization twice a week with the nitrate of mercury; baths.—This treatment was continued until the 6th of March, without any perceptible improvement; she was then cauterized twice a week with Vienna paste solidified, (caustic potass and carbonate of lime,) and injections of the acetate of alum, three times a day, were substituted for those first used. The ulceration began to give way under this treatment, and was nearly well, when, on the 1st of May, she was taken in labour, and delivered of a full grown child. The labour was tedious, but unaccompanied by any unusual occurrence. The ulceration re-appeared after delivery, but gave way to emollient and then astringent injections, and she left cured, on the 6th of July."

"*Severe ulceration of the cervix in a woman two months pregnant; cured by cauterization with potassa fusa.*

"Louise Lejeune, aged twenty-nine, two months pregnant, entered St. Lazare on the 28th of February, 1843. She was delivered of a full grown child a year previous, and miscarried five months ago, at two months and a half, without any assignable cause. On the cervix an ulceration, covered with fungous, vegetating granulations, three quarters of an inch in diameter; considerable inflammatory induration and hypertrophy of the urine neck, which is of a violet hue. Constant pain in the hypogastrium. Abundant mucoso-purulent discharge.

"On the 1st of March, the ulcerated surface was cauterized with the solidified potassa fusa. The cauterization was afterwards repeated once every week. A ball of lint, spread over with mercurial ointment, was daily applied to the ulceration; and vaginal injections of a decoction of walnut leaves were used three times a day.

"On the 27th of March, the granulations had lost their fungous character, and the deep violet hue which they at first presented, was less marked.

"On the 1st of April, the nitrate of silver was substituted to the potassa fusa, the treatment being otherwise the same; and on the 15th of May she left, cured. The cervix was still red where the ulceration had existed. All pain had disappeared."

Passing over the Chapters on syphilitical ulcerations, we come to those most important to our readers, on the treatment of ulcerations of the uterine neck.

"INFLAMMATION OF THE CERVIX WITHOUT ULCERATION.

"To obtain the full effect of the injections, the patient should recline on the side of a bed, or of a lounging chair, elevating the pelvis, so as for the vagina to form an inclined plane, of which the cervix is the most dependent point. The vagina thus retains the injected fluid, like a vase; it penetrates gradually into every part, and remaining in contact with the inflamed tissues for a few minutes, exercises a decided influence on them.

"Not only is it possible to treat successfully non-ulcerated inflammation of the cervix without the introduction of the speculum, merely by emollient and astringent injections, rest, and attention to general health, but even slight ulcerations unaccompanied by general inflammatory hypertrophy, will give way under the same treatment. In order to establish this fact, I have repeatedly, after ascertaining with the speculum the presence of a superficial ulceration, treated the patient merely as described, without touching the ulcerated surface with caustic, and have found, in many instances, the inflammation diminish, and the ulceration decrease, and at last cicatrize. It is only, however, in cases of very slight ulceration, unaccompanied by general hypertrophy, that rest combined with emollient and astringent injection, succeeds; and even in these cases the treatment is much more tedious and uncertain than if cauterization of the ulcerated surface is at once resorted to and repeated as required."

"INFLAMMATION OF THE CERVIX WITH ULCERATION.

"In order to apply a fluid caustic, the following plan should be resorted to:—A small thin stick, about a foot in length, having been chosen, is formed into a brush, by inserting between its divided extremities a little wool, lint, or old linen, which is then fastened by a few turns of thread. These little brushes may be made *ex tempore*, and being of no value, can be thrown away when they have been used. The brush, having been introduced into the acid, should be pressed against the rim of the bottle, in order that it may be merely moistened with the caustic, and then thrown over the ulcerated surface. A little water must then be injected into the speculum before withdrawing it, in order to absorb any super-abundant acid. This precaution is not absolutely necessary, if care has been taken not to use too moist a brush. Owing to the powerful cauterizing properties of acid, it is perhaps, as well, however, for a person unaccustomed to the treatment of these diseases, to adopt the precaution. In that case a syringe, holding about half-a-pint of water, should be used, and the water injected before the speculum is withdrawn. By changing the position of the pelvis, the fluid may afterwards be easily made to fall into the basin. I often merely pour a little olive oil into the speculum; the effect obtained is the same. When the nitrate of silver is employed, no precaution is necessary. The contact of the uncombined caustic with the neighboring parts, nearly always inflamed, can only be productive of benefit. In cauterizing the cervix, the speculum must be *firmly* applied to the parts, so as to protect the vagina from the action of the caustic."

The diseases which affect the uterine system are perhaps of more importance to physicians of the Western States, than to those located elsewhere, for the prevalence and severity of these affections here is proverbial.

The fact that nearly all the women in the new states are young and inexperienced, and subject to all the causes of such affections, and in addition to many hardships and exposures and the influence which produces periodical diseases, as ague, &c, will sufficiently account for their frequency. For it is not to be forgotten that engorgements of the uterine body, or neck speedily affect the general system; and in their turn are aggravated by whatever deranges the functions of the system. It is perhaps a fault of the work of which we have been giving some extracts, that it tends to fix the attention too exclusively upon the local treatment, to the neglect of equally important general remedies. It is not improbable, also, that the entire neglect of mechanical means for giving support to the uterus when displaced is a defect. For although the displacement may and be, in most cases undoubtedly is, produced by inflammation, it is no less true that it remains after inflammatory action is removed, and requires appropriate treatment. Experience, moreover, which has shown the general inutility of pessaries and supports has shown their value in some cases.

In short while we think that this work will render a service to the public, by drawing their attention to a class of affections but little known and badly treated, it is necessary to guard against adopting too exclusively the views of treatment therein set forth. D. B.

